# Underrepresentation of women in cardiovascular disease clinical Trials—What’s in a Name?

**DOI:** 10.1016/j.ijcha.2024.101547

**Published:** 2024-11-08

**Authors:** A.E. Spiering, A.M.L.N. van Ommen, J.E. Roeters van Lennep, Y. Appelman, K. Reue, N.C. Onland-Moret, H.M. den Ruijter

**Affiliations:** a Laboratory of Experimental Cardiology, University Medical Center Utrecht, Utrecht University, Utrecht, the Netherlands; b Department of Human Genetics, David Geffen School of Medicine, University of California Los Angeles, Los Angeles, United States; c Department of Internal Medicine, Erasmus Medical Center, Rotterdam, the Netherlands; d Department of Cardiology, Heart Center, Amsterdam UMC – Vrije Universiteit Amsterdam, Amsterdam Cardiovascular Sciences, Amsterdam, the Netherlands; e Department of Epidemiology, Julius Center for Health Sciences and Primary Care, University Medical Center Utrecht, Utrecht University, Utrecht, the Netherlands

**Keywords:** Cardiovascular disease, Clinical trials, Female representation, Trial acronym, Female authorship, Perceived gender

## Abstract

**Background:**

Cardiovascular disease is the leading cause of death in women worldwide. Yet, women are often underrepresented in cardiovascular clinical trials. Trial characteristics may influence the participation of women. For instance, trials are often entitled with an acronym, which might be perceived as gendered. We aimed to investigate if the perceived gender of the acronym and other trial characteristics affect the representation of female patients in cardiovascular trials.

**Methods:**

We searched ClinicalTrials.gov for randomized controlled trials in cardiovascular disease named with an acronym. Cardiovascular patients (n = 148) scored the perceived gender of the acronym of 148 identified trials. Prevalence ratios (PR) were calculated with Poisson regression to link trial characteristics to representation of female patients in the trials.

**Results:**

In 62 % of trials, female patients were underrepresented relative to the disease population. There was no improvement over time in proportion of trials with adequate representation. A third of acronyms was classified as gendered. The perceived gender did not affect representation of female patients (PR 1.01; 95% CI 0.95 – 1.08; P = 0.68). A woman as first and/or last author (PR 1.22; 95% CI 1.07 – 1.38; P = 0.002) and recruitment in an outpatient setting (PR 1.15; 95% CI 1.02 – 1.29; P = 0.01) were associated with a higher prevalence of adequate representation of female patients.

**Conclusions:**

Representation of female patients in cardiovascular trials does not depend on the perceived gender of the trial acronym but is improved in trials under female leadership in out-patient settings. Our findings may direct efforts towards increasing representation of female patients in cardiovascular trials.

## Graphical abstract

**Figure:**
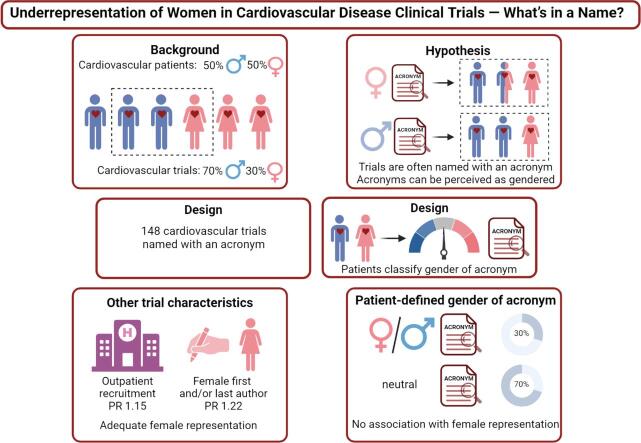

## 1 Introduction

Cardiovascular disease is the leading cause of death in women worldwide, and approximately half of cardiovascular patients are women [1], [2]. Regardless, women are historically poorly represented in clinical trials of cardiovascular disease [3]. Since sex differences exist in disease etiology and drug pharmacokinetics [4], [5], representation of female patients in clinical research is essential to assess if sex differences occur in efficacy and adverse reactions [6], [7], [8]. For instance, in 325 randomized trials of cardiovascular disease between 1997 and 2009, only 30 % of the participants were women [9].

Previously identified obstacles for underrepresentation of female patients and include harm perception as well as socioeconomic, financial and logistical barriers of female patients [1], [10]. Additionally, trial design factors such as inclusions in tertiary centers where women are less likely to be referred to as well as eligibility criteria seem to play a role [11], [7], [11], [12]. Clinical trials are often labeled with an acronym [13], [14] to attract attention to the trial as well as being a reference in the medical community [14], [15]. For instance, the acronym EMPEROR-Reduced [16], a large empagliflozin trial, may be perceived as masculine. Trials could therefore be perceived as feminine or masculine. Gendered words can carry assumptions: Hurricanes with a feminine name are perceived as less dangerous than hurricanes with a masculine name [17] and scientific awards named after women are more frequently awarded to female scientists [18].

Here, we assessed the relation between trial characteristics, including the perceived gender of trial acronym, and enrollment of female patients in cardiovascular trials. We hypothesized that a perceived gender of the trial acronym (i.e., perceived as feminine or masculine) influences the enrollment of female patients in the sense that especially masculine trial acronyms may be associated with underrepresentation of female patients.

## 2 Methods

### 2.1 Data Sources and study selection

We searched ClinicalTrials.gov for registered cardiovascular clinical trials on September 5, 2022. The umbrella term “Cardiovascular Diseases” was combined with more confined terms such as “Heart Failure” and “Hypertension”. To increase homogeneity, we limited the search to drug trials and excluded trials testing diagnostic methods, devices and lifestyle or health service interventions. After removal of duplicates, we excluded studies with less than 100 participants, or were not randomized controlled trials or those that limited their recruitment to children or only one sex (Supplementary Figure 1). A complete overview of advanced search settings is listed in Supplementary Table 1.

Primary outcome publications corresponding to the registration on ClinicalTrials.gov were identified via PubMed. We excluded records if (1) the trial had no acronym; (2) the results were not published in a peer-reviewed journal; (3) the disease type was not one of 8 pre-identified cardiovascular diseases (acute coronary syndrome, arrhythmia, coronary heart disease or atherosclerosis, heart failure, hypertension, pulmonary hypertension, stroke or a combination of these diseases). Records were screened by A.S. and 10 % were independently verified by A.v.O.

### 2.2 Data extraction

Extracted trial characteristics included trial acronym, disease and drug type, the number of participants, mean age and the number and percentage of women included. Additionally, we recorded the presence of sex-specific eligibility criteria, the recruitment setting the region of the coordinating center, funding type, and the journal and year of publication. Finally, we noted the name of the first and last author and determined their gender through manual online search of author names in combination with institutional names. Sources included pronoun descriptors and photographs used on institutional websites or professional networking websites. Data extraction was performed by A.S.

### 2.3 Categorization of acronyms

Trial acronyms were divided into three categories that were based roughly on the classification of trial acronyms proposed by Berkwits et al [13]. Acronym categories included associative, descriptive and without meaning. We considered an acronym (1) associative if it was a given name, a profession, a human characteristic, a color, or other emotionally associative term; (2) descriptive if it was a verb, an object, or a non-associative concept; or (3) without meaning if it was an abbreviation, initialism, neologism or homonym (Supplementary Figure 1). For instance, we categorized the acronyms CANVAS and OPTIC as descriptive, and EASEGO and MOXAF as without meaning. For further analysis, we considered only trials with associative acronyms as we considered those more likely to be perceived as gendered. Categorization of all acronyms was independently conducted by A.S. and A.v.O., and disagreements were resolved by discussion.

### 2.4 Survey to determine Masculinity-Femininity Index

We conducted an online survey among a panel of 148 cardiovascular patients that were recruited via a private Facebook group linked to a Dutch organization for patients with cardiovascular disease, and they responded to the survey (year 2022). We recorded patient self-reported gender (male, female, other) and age. Of 148 included trials, there were 35 trials with shared or similar acronyms (e.g. CHARM-Preserved and CHARM-Alternative). For these cases, we represented the overlapping acronyms by using the root form in our survey, omitting any suffixes, prefixes or numbers(e.g., CHARM). As a neutral control, we added 21 acronyms to the survey that we categorized as “descriptive”. Each patient was presented 50 random acronyms to evaluate.

Patients evaluated trial acronyms for their perceived gender using a 1 to 5 Likert scale (1 = feminine, 2 = a bit feminine, 3 = neutral, 4 = a bit masculine, 5 = masculine). We averaged the perceived gender scores to derive the continuous Masculinity-Femininity Index per trial acronym, as described previously [17]. A score ≥ 4 was considered as masculine and a score ≤ 2 as feminine, respectively. Inter-rater reliability of scores was assessed with Krippendorff’s alpha test.

### 2.5 Participation-to-Prevalence Ratio

For each trial, the Participation-to-Prevalence Ratio [3] was computed by dividing the percentage of women among the trial participants by the percentage of women among the disease population. The corresponding proportion of women in each disease population was obtained from large epidemiological studies (Supplementary Table 2). A Participation-to-Prevalence Ratio of < 0.8 or > 1.2 indicates that female patients are underrepresented or overrepresented relative to the disease population, respectively [3]. 4The Participation-to-Prevalence Ratio was dichotomized, where a Participation-to-Prevalence Ratio between 0.8 and 1.2 was defined as adequate, a Participation-to-Prevalence Ratio below 0.8 as underrepresentation and a Participation-to-Prevalence Ratio above 1.2 as overrepresentation.

### 2.6 Statistical analysis

Data are presented as mean and standard deviation (± SD) for normal continuous variables, median and interquartile range [IQR] for non-normal continuous variables, and numbers (percentages) for categorical variables..

First, we calculated the Masculinity-Femininity Index based on survey results. We stratified female participation outcomes by the perceived gender of the trial acronyms, and plotted the Masculinity-Femininity Index on the Participation-to-Prevalence Ratio. We associated trial characteristics, including the Masculinity-Femininity Index, to the prevalence of having adequate female participation using Poisson regression with robust variance estimation. Underrepresentation of female patients (Participation-to-Prevalence Ratio < 0.8) was the reference for prevalence ratios (PR). Both univariable and multivariable models were performed. Variables in the multivariable model were selected based on a p-value < 0.15 in the univariable model.

We performed two sensitivity analyses regarding Masculinity-Femininity Index by stratifying by female and male responders to the survey, and by excluding the trials with a neutral acronym. All reported p-values are two-tailed, and the level of significance is set at alpha < 0.05. Data was analyzed using R statistical software (version 4.2.3).

## 3 Results

3.1Trial Characteristics.

In total, we identified 10,695 trials of which 5,817 remained for screening after removal of duplicates. We categorized 574 trial acronyms from eligible trials. 148 trial acronyms were classified as associative and included in the data extraction and analysis process (Supplementary Figure 1). Trial characteristics are reported in Table 1. The 489,236 participants had a mean age of 63 (SD 6.2) years and 146,299 (30 %; range = 8.3 % to 83.6 %) were women. Most trials were multicenter (95 %), sponsored by industry (75 %) and recruited their patients at an outpatient setting (61 %) in North America (48 %). Sex-specific eligibility criteria were present in 55 % of trials, and sex-stratified results were reported in 45 % of publications. The first and last authors were predominantly male (89 % and 93 %, respectively).

**Table 1:** Characteristics of 148 cardiovascular drug trials included in this study.

**Trial characteristics**	**No. (%) of trials**
*General characteristics*	
Disease	
CHD/Atherosclerosis	37 (25.0)
Heart Failure	28 (18.9)
Hypertension	22 (14.9)
Acute Coronary Syndrome	21 (14.2)
Arrhythmias	13 (8.8)
Multiple	13 (8.8)
Pulmonary Arterial Hypertension	7 (4.7)
Stroke	7 (4.7)
Therapeutic class	
Multiple	21 (14.2)
Anti-platelet therapy	21 (14.2)
ACE-inhibitors, ARBs and RIs	17 (11.5)
Statins	12 (8.1)
Vasodilators	11 (7.4)
Lipid-lowering agents (non-statins)	9 (6.1)
Anti-arrhythmic agents	7 (4.7)
Diuretics	6 (4.1)
Direct oral anticoagulants	6 (4.1)
SGLT2-inhibitors	5 (3.4)
Calcium channel blockers	4 (2.7)
Beta blockers	3 (2.0)
Other	26 (17.6)
Number of participants	
100 – 300	38 (25.7)
300 – 1000	42 (28.4)
1000–––3000	28 (18.9)
3000–––27564	40 (27.0)
Age based on mean age of participants^a^	63.2 (± 6.2)
Percentage of female participants	30.7 [23.9 – 41.5]
Recruitment	
Outpatient	90 (60.8)
Inpatient	58 (39.2)
Sex-specific eligibility criteria present	81 (54.7)
Sex-stratified results present	67 (45.3)
*Publication*	
Year of publication	
1992 – 2008	40 (27.0)
2009 – 2012	31 (20.9)
2013 – 2018	39 (26.4)
2019 – 2022	38 (25.7)
Journal	
NEJM	45 (30.4)
JAMA	13 (8.8)
Lancet	11 (7.4)
Circulation	10 (6.8)
JACC	6 (4.1)
Other	63 (42.6)
Female first author gender	16 (11.0)
Female last author gender	10 (6.8)
*Administrational*	
No. of centers	
Single center	7 (4.7)
Multicenter	141 (95.3)
Region of the coordinating center	
North America	71 (48.0)
Europe	57 (38.5)
Asia + MENA	20 (13.6)
Funding b	
Industry	108 (75.0)
Public	19 (13.2)
Mixed	17 (11.8)
Year of start recruitment^b^	
1984–––2003	29 (21.6)
2004 – 2007	35 (26.1)
2008 – 2013	36 (26.9)
2014 – 2020	34 (25.4)

Abbreviations: ACE, angiotensin-converting enzyme; ARB, angiotensin receptor blockers; CHD, coronary heart disease; JACC, Journal of the American College of Cardiology; JAMA, Journal of the American Medical Association; MENA, Middle East North Africa; NEJM, New England Journal of Medicine; RI, renin inhibitors; SGLT2, sodium-glucose transport protein 2.

a 17 trials reported median age, which was used as a substitute for mean age in this table.

b values do not add up to 148 due to missing data.

3.3Masculinity-Femininity Index.

A total of 148 respondents (68 % female, median age 61 y [IQR, 54 – 66]) rated trial acronyms for their perceived gender. On average, each acronym was rated by 32 individuals (range: 25 – 36). The mean Masculinity-Femininity Index was 3.17 ± 0.87 (Supplementary Figure 2). Only 26 (17.6 %) and 17 (11.5 %) trials were perceived as masculine (Masculinity-Femininity Index ≥ 4) or feminine (Masculinity-Femininity Index ≤ 2), respectively. Examples of trial acronyms perceived as feminine, masculine or neutral are VICTORIA (Masculinity-Femininity Index 1.15), ADONIS (Masculinity-Femininity Index 4.53), ACADEMY (Masculinity-Femininity Index 3.15). The neutral descriptive acronyms that were included as control were evaluated with a mean Masculinity-Femininity Index of 3.14 ± 0.20 (Supplementary Figure 3). On average, the 100 female respondents (68 %) gave similar Masculinity-Femininity Index scores as the 48 male respondents (32 %), 3.17 SD 0.95 and 3.14 SD 0.77, respectively (*p* = 0.42). There was poor inter-rater reliability of scores (Krippendorff’s α = 0.42), and this was similar for only female or male raters (Krippendorff’s α = 0.45 and 0.37, respectively). The inter-rater reliability was higher among the 28 trials with a given name as acronym (Krippendorff’s α = 0.71).

### 3.1 Enrollment of female patients

Female patients were underrepresented in 91 of the 148 included trials (i.e. 61.5 % of trials had a Participation-to-Prevalence Ratio < 0.8) (Table 1). Female patients were most frequently underrepresented in trials of arrhythmias (85 %) and acute coronary syndrome (76 %) (Supplementary Figure 4). In trials of pulmonary arterial hypertension, female patients were never underrepresented. No trials in our study overrepresented female patients (Participation-to-Prevalence Ratio > 1.2). Finally, the proportion of trials with adequate representation of female patients (Participation-to-Prevalence Ratio ≥ 0.8) did not increase over time. The proportion of trials with a Participation-to-Prevalence Ratio ≥ 0.8 decreased from 50.0 %, to 38.7 %, 33.3 % and 31.6 % in trials published between 1992 and 2008, 2009 and 2012, 2013 and 2018, and 2019 and 2022, respectively (*p* for trend = 0.059) (Supplementary Figure 5**A**). This trend is similar for the year of start recruitment: the proportion decreased from 48.3 % to 45.7 % to 30.6 % to 29.4 % in trials that started recruitment between 1984 and 2003, 2004 and 2007, 2008 and 2013, and 2014 and 2020, respectively (*p* for trend = 0.109) (Supplementary Figure 5**B**).

### 3.2 Trial acronyms and representation of female patients

**Fig. 1:**
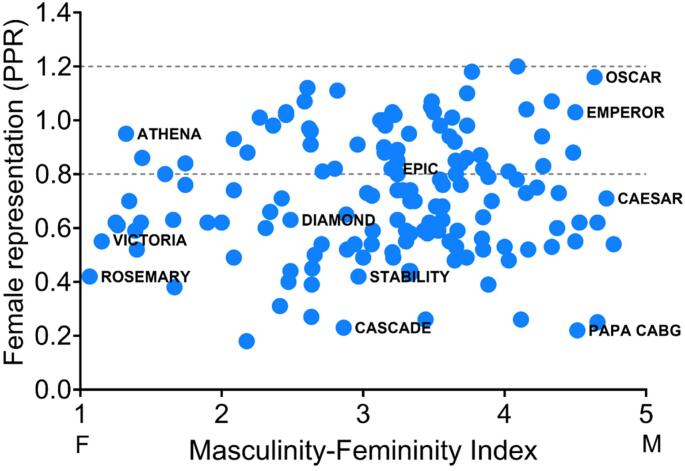
**The relationship between Masculinity-Femininity Index of trial acronym and Participation-to-Prevalence Ratio.** Scatterplot of all 148 trials, of which 11 trials labeled by name. Dotted lines represent Participation-to-Prevalence Ratio cut-off values (female underrepresentation < 0.8 and overrepresentation > 1.2). Scale of Masculinity-Femininity Index: 1 = feminine, 2 = a bit feminine, 3 = neutral, 4 = a bit masculine, 5 = masculine. Abbreviations: PPR, Participation-to-Prevalence Ratio.

The relationship between perceived gender of trial acronyms (Masculinity-Femininity Index) and representation of female patients (Participation-to-Prevalence Ratio) is visualized in the scatterplot in Fig. 1, and stratified by perceived gender in Supplementary Table 3. Masculinity-Femininity Index was not statistically significantly associated with the prevalence of adequate female representation (PR 1.01, 95 % CI 0.95 – 1.08), neither after adjustment for potential confounders (recruitment type, number of participants, female first and/or last authorship and year of publication) that were identified from univariable models (PR 1.03, 95 % CI 0.97 – 1.09). Furthermore, in the sensitivity analyses, there was no association when only using survey outcomes from female or male respondents, or after exclusion of trials with a neutral Masculinity-Femininity Index (Table 2).

**Table 2:** Univariable and multivariable Poisson regression analysis of Masculinity-Femininity Index and adequate female representation.

**All trials (n = 148)**		
	**Model 1 – Univariable**	**Model 2 – Multivariable**
	Prevalence Ratio (95% CI)	Prevalence Ratio (95% CI)
MFI (all raters)	1.01 (0.95 – 1.08)	1.03 (0.97 – 1.09)
MFI (female raters only)	1.01 (0.96 – 1.07)	1.03 (0.97 – 1.09)
MFI (male raters only)	1.00 (0.94 – 1.08)	1.03 (0.96 – 1.10)
**Trials with a feminine or masculine acronym only (n = 43)**		
	**Model 1 – Univariable**	**Model 2 – Multivariable**
	Prevalence Ratio (95% CI)	Prevalence Ratio (95% CI)
MFI (all raters)	1.03 (0.96 – 1.10)	1.03 (0.97 – 1.09)
MFI (female raters only)	1.02 (0.96 – 1.09)	1.03 (0.97 – 1.09)
MFI (male raters only)	1.05 (0.96 – 1.14)	1.03 (0.96 – 1.1

Abbreviations: MFI, Masculinity-Femininity Index. Adequate female representation is defined as a Participation-to-Prevalence Ratio between 0.8 and 1.2. A Participation-to-Prevalence Ratio < 0.8 is the definition of female underrepresentation and serves as reference category.

The multivariable model is adjusted for recruitment type, number of participants, female first and/or last author and year of publication.

### 3.3 Other trial characteristics in relation to representation of female patients

Next, we investigated whether trial characteristics other than perceived gender of trial acronym were related to adequate representation of female patients. Trial characteristics are shown stratified by representation of female patients in Supplementary Table 4. We performed univariable and multivariable analyses using Poisson regression with robust variance estimation (Table 3). After adjustment for potential confounders, trials recruiting in an out-patient setting (PR 1.15, 95% CI 1.02– 1.29) had an increased prevalence of adequate representation of female patients. Also trials with a woman as a first and/or last author (PR 1.22, 95% CI 1.07 – 1.38) were statistically significantly associated with prevalence of adequate representation of female patients (Table 3).

**Table 3:** Univariable and multivariable Poisson regression analysis of trial characteristics and adequate female representation.

	**Model 1 – Univariable**	**Model 2 – Multivariable**
	Prevalence Ratio (95% CI)	Prevalence Ratio (95% CI)
Out-patient recruitment	**1.17 (1.04 – 1.31)**	**1.15 (1.02 – 1.29)**
Sex-dependent eligibility criteria	0.99 (0.89 – 1.12)	1.05 (0.94 – 1.17)
Reporting of sex-stratified results	0.98 (0.88 – 1.10)	1.01 (0.89 – 1.15)
Female first and/or last author	**1.23 (1.08 – 1.40)**	**1.22 (1.07 – 1.38)**
Number of participants		
100 – 300	Ref	Ref
300 – 1000	1.08 (0.93 – 1.25)	1.05 (0.91 – 1.22)
1000–––3000	1.08 (0.92 – 1.28)	1.03 (0.88 – 1.22)
3000–––27564	0.96 (0.82 – 1.11)	0.93 (0.80 – 1.08)
Region		
North America	Ref	Ref
Asia + MENA	0.98 (0.87 – 1.11)	0.97 (0.81 – 1.17)
Europe	0.92 (0.77 – 1.10)	0.99 (0.88 – 1.11)
Funding		
Industry	Ref	Ref
Public	0.93 (0.79 – 1.11)	0.99 (0.82 – 1.18)
Mixed	0.96 (0.80 – 1.15)	0.97 (0.79 – 1.18)
Year of publication†		
1992 – 2008	Ref	Ref
2009 – 2012	0.93 (0.79 – 1.09)	0.89 (0.75 – 1.04)
2013 – 2018	0.89 (0.76 – 1.03)	0.90 (0.78 – 1.04)
2019 – 2022	0.88 (0.75 – 1.02)	0.89 (0.76––1.04)
Year of start recruitment^i^		
1984–––2003	Ref	Ref
2004 – 2007	0.98 (0.83 – 1.16)	1.02 (0.83 – 1.24)
2008 – 2013	0.88 (0.74 – 1.04)	0.90 (0.70 – 1.15)
2014 – 2020	0.87 (0.73 – 1.04)	0.84 (0.60 – 1.19)
Age based on mean age of patients	0.99 (0.98 – 1.00)	1.00 (0.99 – 1.01)
Journal		
Other	Ref	Ref
Circulation	0.98 (0.78 – 1.24)	0.98 (0.77 – 1.24)
JACC	1.05 (0.79 – 1.39)	1.05 (0.81 – 1.37)
JAMA	1.02 (0.83 – 1.25)	0.96 (0.78 – 1.18)
Lancet	0.96 (0.76 – 1.20)	0.92 (0.69 – 1.23)
NEJM	0.90 (0.79 – 1.03)	0.91 (0.74 – 1.10)

Abbreviations: JACC, Journal of the American College of Cardiology; JAMA, Journal of the American Medical Association; MENA, Middle East North Africa; NEJM, New England Journal of Medicine. A Participation-to-Prevalence Ratio < 0.8 is the definition of female underrepresentation and serves as reference category.

The multivariable model is adjusted for recruitment type, number of participants, female first and/or last author and year of publication.

## 4 Discussion

In our study, the perceived gender of the acronym does not affect representation of female patients. However, female authorship and outpatient recruitment are associated with higher prevalence of adequate female representation relative to the disease population.

### 4.1 Underrepresentation of female patients

We used the Participation-to-Prevalence Ratio as an outcome measure as opposed to uncorrected enrollment percentages, as these do not account for sex differences in disease prevalence.

In our study, 29.9 % of 489,236 trial participants were women and as many as 61.5 % of trials underrepresented women. We are not the first to demonstrate and address underrepresentation of female patients in cardiovascular trials [3], [7], [9], [10], [12], [19], [20], [21], [22], [23]. While regulatory and funding agencies have initiated efforts to address this [11], we show no improvement in inclusion ratios for trials published between 1992 and 2022 as the proportion of cardiovascular trials with adequate representation of female patients in fact showed a non-significant reduction from 50.0 % to 31.6 %. In practice, the publication date is often years behind the start of recruitment, so it may not reflect the most recent efforts. However, we observed a similar trend in recruitment start times, with a non-significant reduction from 48.3 % to 29.4 % between 1984 and 2020. In line with our findings, most studies using prevalence-adjusted metrics did not report an improvement over time [3], [7], [9], [10], [12], [20], [22].

### 4.2 Female authorship

Notably, our analyses highlight a critical pattern: trials with women in the first and/or last authorship position had increased prevalence of adequate female enrollment. the percentage of female first and last authors in our included trials was low: only 11.0 % of first and 6.8 % of last authors of publications in our study were women, in line with numbers reported by others [1], [7], [11], [24], [25], [26], [27], [28], [29]. These studies are more likely to consider sex as a variable in their analysis [7], [27], [28], [30] and to report sex-specific differences in trial flow, treatment efficacy or adverse events [31]. Bibliometric analyses showed that cardiology articles with women in last authorship positions are more likely to have women as first or middle authors, possibly reflecting the influence of female role models [24], [28], [33], [34]. We assume that the studies with female first or last authorship are also led by these same women.Female leaders may be able to design trials that are more accommodating for female participants [28] and greater visibility of women in trial leadership positions may increase the recruitment of female patients [24], [32]. Thus, advancing female representation among trial leadership may be a starting point to improve female representation among trial participants.

### 4.3 Factors linked to female representation

Our results are consistent with prior publications demonstrating female underrepresentation in cardiovascular trials, summarized in Supplementary Table 5 [3,7,9,10,12,19–23]. For instance, others already reported that recruitment in an outpatient setting was associated with adequate representation of female patients [7]. This may result from sex-related referral bias for trial participation, or be associated to participation barriers experienced by women [24]. We did not find that age of participants adequate and female participation was linked. Most studies reported higher female enrollment in cardiovascular trials with advancing age of patients [9], [20], [22]. Therefore, strategies to improve female enrollment in cardiovascular clinical trials could still consider age.

Female −specific exclusion criteria were present in most studies and rangedfrom excluding pregnant women to excluding women of childbearing potential [31]. These criteria are often implemented to protect a “vulnerable population” and are certainly justified in some cases. Nevertheless, we propose to evaluate the potential exclusion of women with a specific child-bearing status on a trial-by-trial basis [7], [11], [35].

Finally, since we only included trials with an acronym, it is possible that our other findings are not generalizable to trials without an acronym. However, other studies without acronym-specific selection criteria have previously already described female authorship and recruitment type as factors that influence female participation in clinical trials [3], [9], [10], [12], [29].

### 4.4 Trial acronyms

Yet, our survey resulted in the majority (71 %) of the trial acronyms being perceived as gender neutral. The sensitivity analysis conducted with only the gendered acronyms (29 %) did not yield different results. Male and female patients did not score the Masculinity-Femininity Index significantly different, butthe interrater reliability was low (α = 0.42). Low interrater reliability and the gender-neutral perception of trial names may be related to the small proportion (18.9 %) of trial acronyms recognized as given names, which might be more readily perceived as gendered. Indeed, interrater reliability of the Masculinity-Femininity Index was considerably higher for trial acronyms with a given name(α = 0.71)).

Our Participation-to-Prevalence Ratio metric assumes that perceived masculinity and femininity are mutually exclusive and may not capture the full nuance of perceived gender. However, we opted for a simple, narrow and two-dimensional 1 – 5 Likert scale, as we expected that increased survey complexity would result in a lower response rate. Along with the low proportion of given names among the acronyms, these factors likely explain the poor inter-rater reliability observed in our study.

## 5 Study Limitations

We acknowledge that assigning gender and associations to a word is subjective, and our pre-selection of associative trial acronyms may be biased. Secondly, we used Participation-to-Prevalence Ratio as outcome measure in our study. Further breakdown of prevalence by age would have been superior, however such high-quality age-stratified epidemiological data was not available. Since our prevalence estimates were mostly from the United States and do not date from before 2016 (Supplementary Table 2), the prevalence metric may not be accurate for all trials included in our study. In addition, for some diseases, the sex-specific prevalence may fluctuate over time. As a result, we may have over- or underestimated the true Participation-to-Prevalence Ratio in cases where studies were conducted in a different decade from the prevalence estimates used.

### 5.1 Future directions

Evidently, developing effective new strategies incorporating a broad approach and consider societal, socio-economic and logistical barriers for female patients, as well as factors related to trial design and leadership are needed [1]. Possibly, participation of female patients could benefit from outreach initiatives to raise awareness about ongoing trials, personalized approaches including female-targeted information brochures or improved gender diversity among trial leadership [1], [11], [20], [36]. Additionally, researchers that recruit hospitalized patients should be aware that they may need to make a larger effort to enroll enough women.

## 6 Conclusion

Representation of female patients in cardiovascular clinical trials remains poor and does not depend on the perceived gender of the trial acronym. However, representation of female patients is improved in cardiovascular clinical trials under female leadership and when recruitment is performed at outpatient clinics. Our findings may direct efforts towards increasing representation of female patients.

## CRediT authorship contribution statement

**A.E. Spiering:** Writing – original draft, Investigation, Formal analysis. **A.M.L.N. van Ommen:** Writing – review & editing, Investigation, Formal analysis, Conceptualization. **J.E. Roeters van Lennep:** Writing – review & editing. **Y. Appelman:** Writing – review & editing. **K. Reue:** Writing – review & editing. **N.C. Onland-Moret:** Writing – review & editing, Supervision, Methodology. **H.M. den Ruijter:** Writing – review & editing, Supervision, Methodology, Conceptualization.

## Funding

This study was funded by the Dutch Cardiovascular Alliance (2020B004 IMPRESS) and Leducq Foundation (AtheroGEN 22CVD04).

Note: All authors take responsibility for all aspects of the reliability and freedom from bias of the data presented and their discussed interpretation

## Declaration of competing interest

The authors declare that they have no known competing financial interests or personal relationships that could have appeared to influence the work reported in this paper.
