# Supplementary material for: Underrepresentation of women in cardiovascular disease clinical Trials—What’s in a Name?
Source: Int J Cardiol Heart Vasc. 2024 Nov 8;55:101547. doi: 10.1016/j.ijcha.2024.101547 (PMC11584672; doi:10.1016/j.ijcha.2024.101547)
Supplement: Supplementary Data 1 [file mmc1.docx]

**Supplemental Material**

Supplemental Figure 2: Distribution of the Masculinity-Femininity Index.

Supplemental Figure 3: Distribution of the Masculinity-Femininity Index of descriptive trial acronyms.

Supplemental Figure 4: Participation-to-Prevalence Ratio stratified by several trial characteristics.

Supplemental Figure 5: Gradual decrease in representation of female patients over time.

Supplemental Table 1: Advanced search settings at ClinicalTrials.gov.

Supplemental Table 2: Estimation of proportion of women in eight cardiovascular disease categories.

Supplemental Table 3: Measures of Female Participation stratified by Perceived Gender of Trial Acronyms.

Supplemental Table 4: Characteristics of 148 cardiovascular drug trials included in this study stratified by representation of female patients.

Supplemental Table 5: Results from systematic reviews investigating female representation in cardiovascular clinical trials.

References

**Supplemental Figure 1: Flowchart of selection of trials with associative acronyms.**


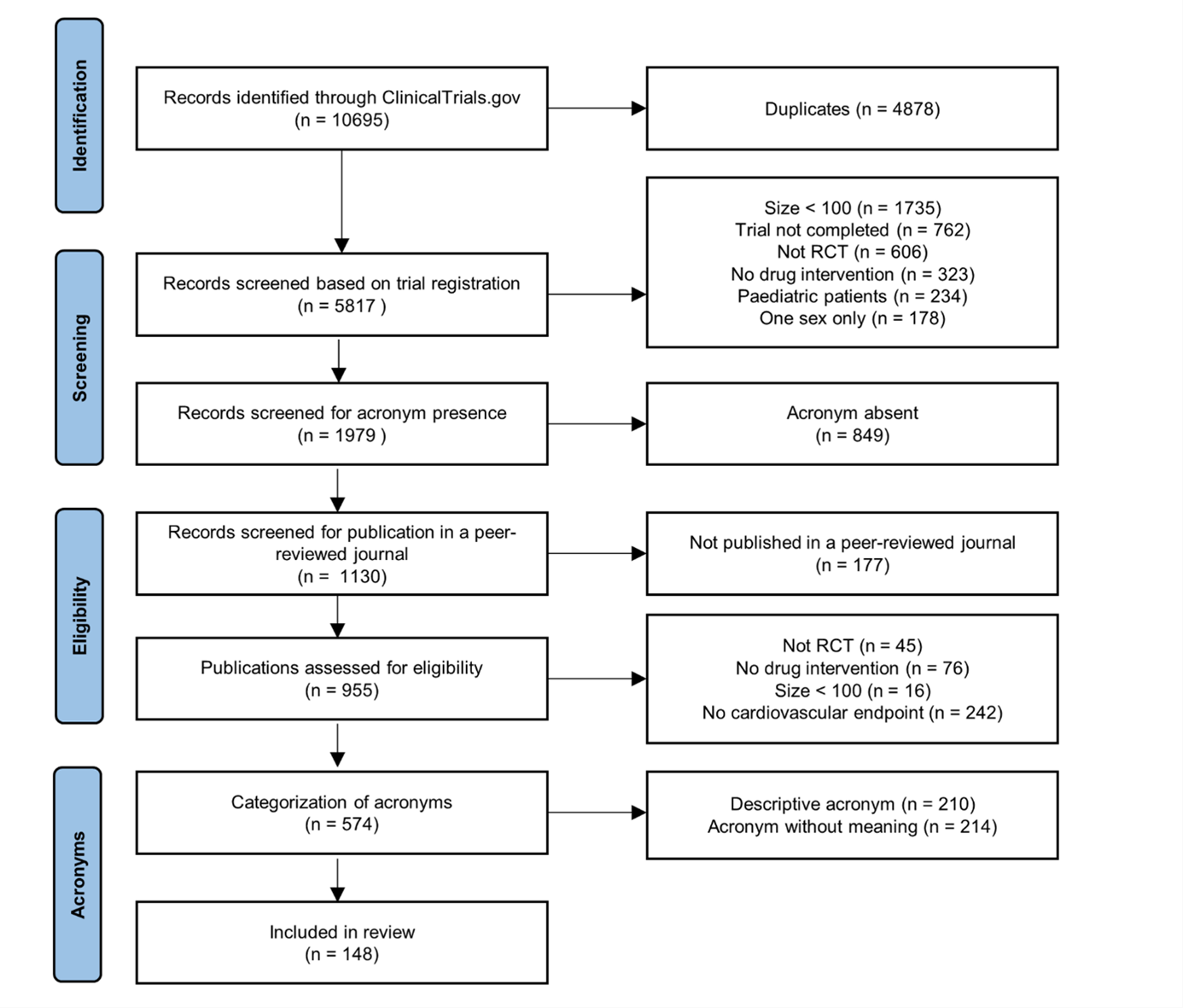


Abbreviation: RCT, randomized controlled trial

**Supplemental Figure 2:** **Distribution of the Masculinity-Femininity Index for 148 trial associative trial acronyms.**


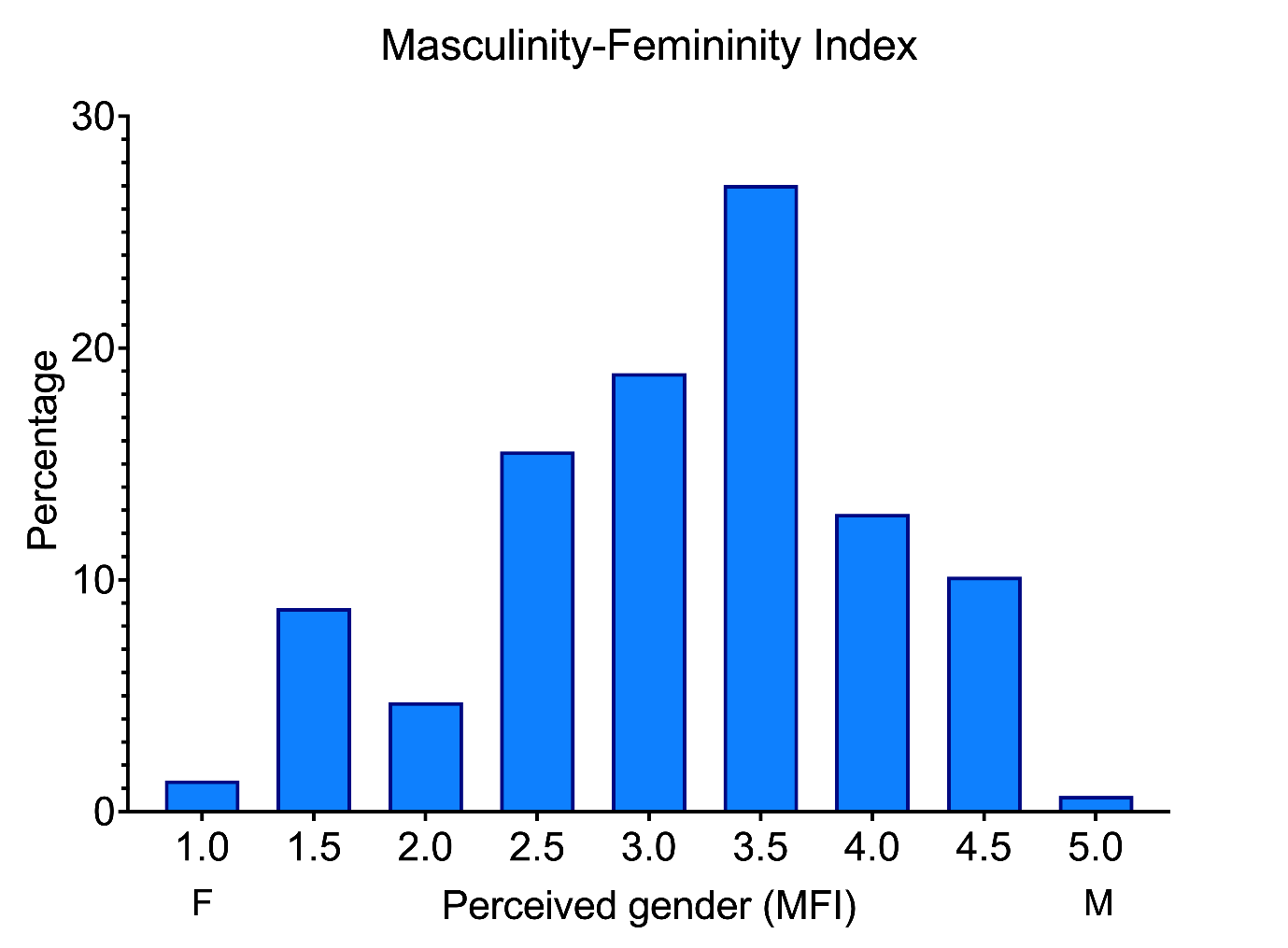


Scale of MFI: 1= feminine, 2= a bit feminine, 3= neutral, 4= a bit masculine, 5= masculine. Abbreviation: MFI, Masculinity-Femininity Index

**Supplemental Figure 3:** **Distribution of the Masculinity-Femininity Index for 21 trial acronyms that served as neutral control.**


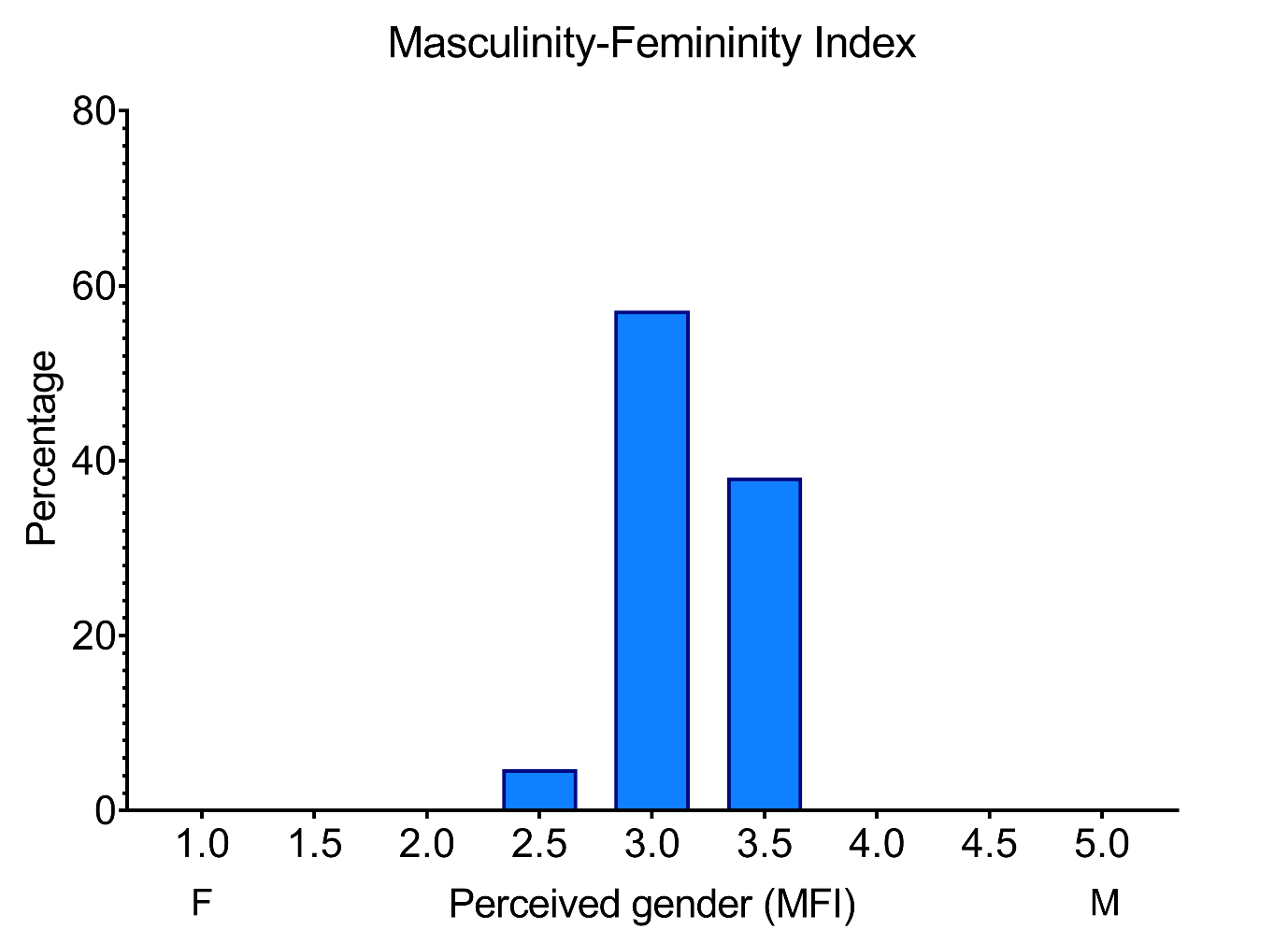


Scale of MFI: 1= feminine, 2= a bit feminine, 3= neutral, 4= a bit masculine, 5= masculine. Abbreviation: MFI, Masculinity-Femininity Index

**Supplemental Figure 4: Participation-to-Prevalence Ratio stratified by several trial characteristics.**


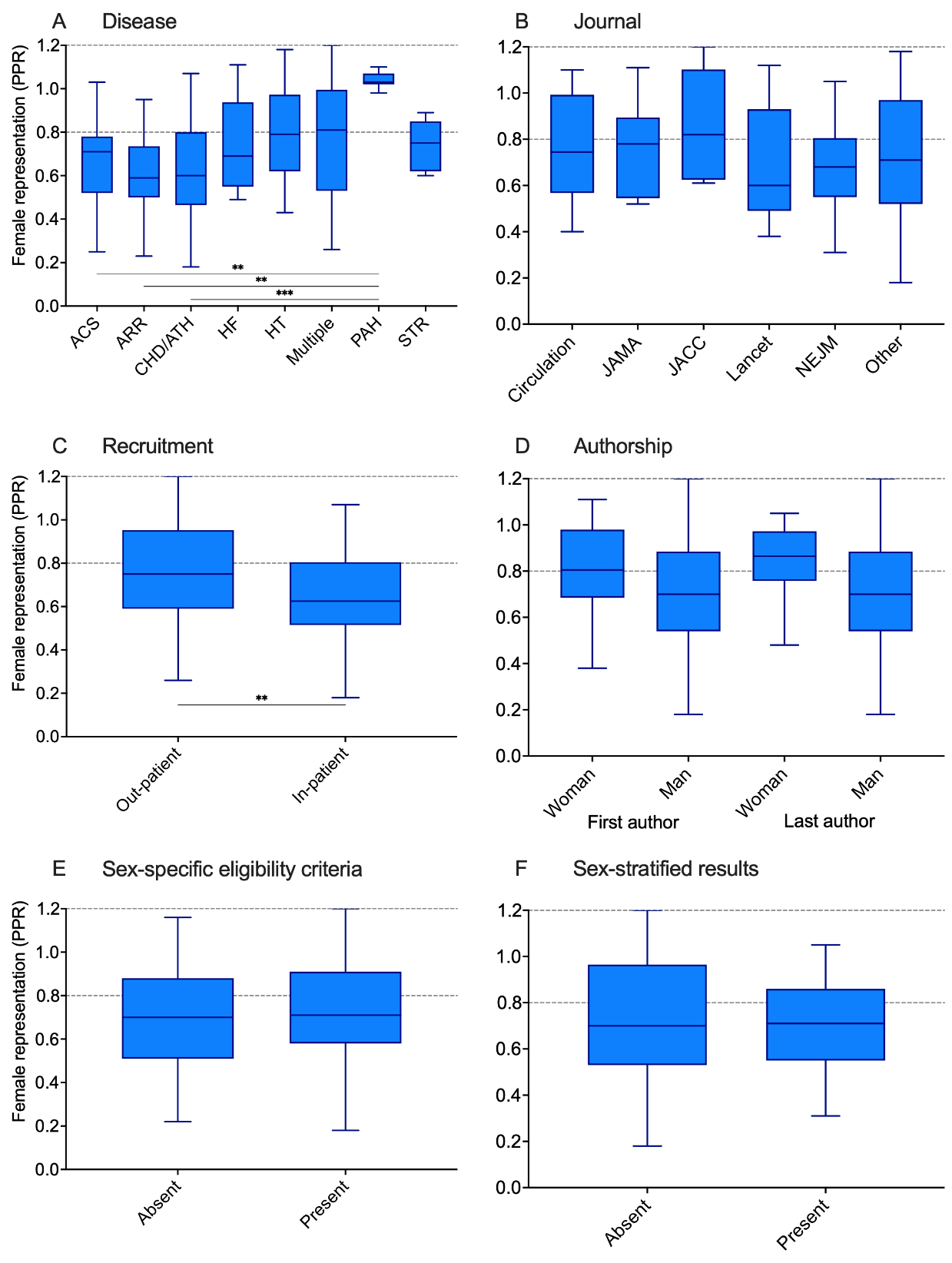


Participation-to-Prevalence Ratio is shown, stratified by (A) disease type; (B) journal; (C) recruitment type; (D) authorship; (E) sex-stratification of results or (F) sex-specific eligibility criteria. Whiskers represent minimum and maximum value. Dotted lines represent Participation-to-Prevalence Ratio cut-off values (female underrepresentation < 0.8 and overrepresentation > 1.2). Mann-Whitney U test and Kruskal-Wallis test with Dunn’s multiple comparison’s correction were used to assess statistical significance between two or more non-normal groups, respectively. Abbreviations: ACS, acute coronary syndrome; ARR, arrhythmias, CHD/ATH, coronary heart disease/atherosclerosis; HF, heart failure; HT, hypertension; JACC, Journal of the American College of Cardiology; JAMA, Journal of the American Medical Association; NEJM, New England Journal of Medicine; PAH, pulmonary arterial hypertension; PPR, participation to prevalence ratio; STR, stroke.

**Supplemental Figure 5: Gradual decrease in representation of female patients over time.**


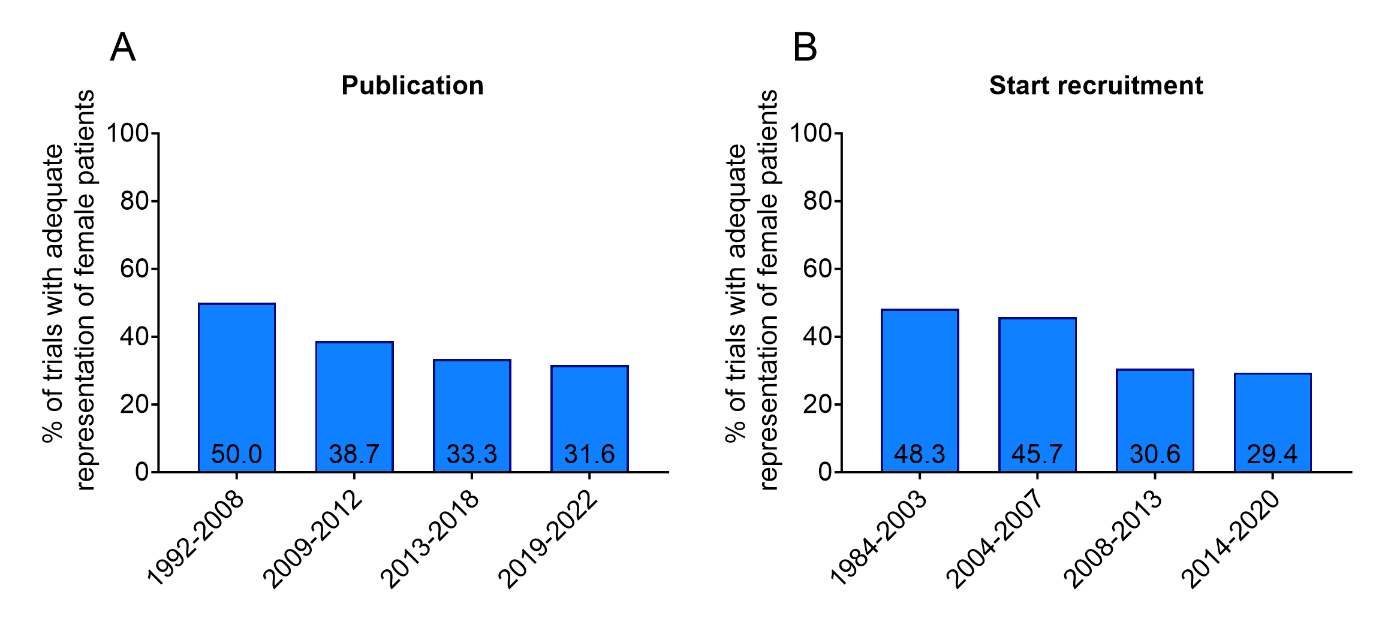


Proportion of trials with adequate female representation is shown for A) time of publication (test for linear trend: p = 0.059) and B) time of start recruitment (test for linear trend: p = 0.109). Female underrepresentation is defined as Participation-to-Prevalence Ratio < 0.8 and overrepresentation as Participation-to-Prevalence Ratio > 1.2.

**Supplemental Table 1: Advanced search settings at ClinicalTrials.gov.**

| **Category** | **Search term** |
| --- | --- |
| **Disease/condition** | “Cardiovascular Diseases” + “Atherosclerosis” + “Arrhythmia” + “Hypertension” + “Heart Failure” + “Pulmonary Hypertension” + “Myocardial Infarction” + “Stroke” + “Coronary Syndrome” + “Coronary Artery Disease” |
| **Study type** | “Interventional” |
| **Status** | “Completed” + “recruiting” + “enrolling by invitation” + “active, not recruiting” |
| **Age** | “Adult” + “older adult” |
| **Sex** | “All” |
| **Intervention/treatment** | “Drug” |
| **Phase** | 3 + 4 |

**Supplemental Table 2:** **Estimation of proportion of women in eight cardiovascular disease categories.**

| **Disease type** | **Proportion of women in the disease population (%)** | **Region** | **Reference** |
| --- | --- | --- | --- |
| Acute coronary syndrome | 34.1% | North America | 39 |
| Arrhythmias | 49.3% | Global | 2 |
| Coronary heart disease/atherosclerosis | 45.0% | North America | 39 |
| Heart failure | 43.3% | North America | 39 |
| Hypertension | 48.1% | North America | 39 |
| Multiple | 52.6% | Global | 2 |
| Pulmonary arterial hypertension | 76.2% | North America | 40 |
| Stroke | 55.6% | Global | 2 |

**Supplemental Table 3: Measures of Female Participation stratified by Perceived Gender of Trial Acronyms.**

|  | **All trials**  (n=148) | **Feminine acronym**  (n=17) | **Neutral acronym** (n=105) | **Masculine acronym**  (n=23) |
| --- | --- | --- | --- | --- |
| Percentage of female participants | 30.7 [23.9 – 41.5] | 30.8 [23.9 – 35.82] | 30.62 [23.91 – 42.20] | 32.12 [23.43 – 42.16] |
| Participation-to-Prevalence Ratio | 0.71 (± 0.23) | 0.65 (± 0.15) | 0.72 (± 0.23) | 0.71 (± 0.27) |
| Proportion of trials underrepresenting female patients | 61.5% | 76.5% | 58.1% | 65.4% |

Feminine acronym is defined as Masculinity-Femininity Index ≤2, neutral acronym as Masculinity-Femininity Index >2 and <4 and masculine acronym as Masculinity-Femininity Index ≥4. Underrepresentation of female patients is defined as a Participation-to-Prevalence Ratio <0.8. Non-normal data is represented as median [IQR].

**Supplemental Table 4:** **Characteristics of 148 cardiovascular drug trials included in this study stratified by representation of female patients.**

|  | | **No. (%) of trials with underrepresentation of female patients** | **No. (%) of trials with adequate representation of female patients** |
| --- | --- | --- | --- |
| *General characteristics* |  |  |  |
| Disease |  |  |  |
| CHD/Atherosclerosis | 27 (73.0) | 10 (27.0) |  |
| Heart Failure | 16 (57.1) | 12 (42.9) |  |
| Hypertension | 11 (50.0) | 11 (50.0) |  |
| Acute Coronary Syndrome | 16 (76.2) | 5 (23.8) |  |
| Arrhythmias | 11 (84.6) | 2 (15.4) |  |
| Multiple | 6 (46.1) | 7 (53.9) |  |
| Pulmonary Arterial Hypertension | 0 (0.0) | 7 (100.0) |  |
| Stroke | 4 (57.1) | 3 (42.9) |  |
| Therapeutic class |  |  |  |
| Multiple | 11 (52.4) | 10 (47.6) |  |
| Anti-platelet therapy | 18 (85.7) | 3 (14.3) |  |
| ACE-inhibitors, ARBs and RIs | 11 (64.7) | 6 (35.3) |  |
| Statins | 6 (50.0) | 6 (50.0) |  |
| Vasodilators | 1 (9.1) | 10 (90.9) |  |
| Lipid-lowering agents (non-statins) | 8 (88.9) | 1 (11.1) |  |
| Anti-arrhythmic agents | 5 (71.4) | 2 (28.6) |  |
| Diuretics | 4 (67.7) | 2 (33.3) |  |
| Direct oral anticoagulants | 4 (67.7) | 2 (33.3) |  |
| SGLT2-inhibitors | 3 (60.0) | 2 (40.0) |  |
| Calcium channel blockers | 3 (75.0) | 1 (25.0) |  |
| Beta blockers | 2 (67.7) | 1 (33.3) |  |
| Other | 15 (57.7) | 11 (42.3) |  |
| Number of participants |  |  |  |
| 100 – 300 | 26 (65.0) | 14 (35.0) |  |
| 300 – 1000 | 15 (53.6) | 13 (46.4) |  |
| 1000 - 3000 | 23 (54.8) | 19 (45.2) |  |
| 3000 - 27564 | 27 (71.1) | 11 (28.9) |  |
| Age based on mean age of patientsˣ | 63.8 (± 5.0) | 62.3 (± 7.6) |  |
| Recruitment |  |  |  |
| Outpatient | 43 (74.1) | 15 (25.9) |  |
| Inpatient | 48 (53.3) | 42 (46.7) |  |
| Sex-specific eligibility criteria present | 50 (61.7) | 31 (38.3) |  |
| Sex-stratified results present | 42 (62.7) | 25 (37.3) |  |
| *Publication* |  |  |  |
| Year of publication |  |  |  |
| 1992 – 2008 | 20 (50.0) | 20 (50.0) |  |
| 2009 – 2012 | 19 (61.2) | 12 (38.8) |  |
| 2013 – 2018 | 26 (67.7) | 13 (33.3) |  |
| 2019 – 2022 | 26 (68.4) | 12 (31.6) |  |
| Journal |  |  |  |
| NEJM | 32 (71.1) | 13 (28.9) |  |
| JAMA | 7 (53.8) | 6 (46.2) |  |
| Lancet | 7 (63.6) | 4 (36.3) |  |
| Circulation | 6 (60.0) | 4 (40.0) |  |
| JACC | 3 (50.0) | 3 (50.0) |  |
| Other | 36 (57.1) | 27 (42.9) |  |
| Female first author | 7 (43.4) | 9 (56.6) |  |
| Female last author | 2 (20.0) | 8 (80.0) |  |
| *Administrational* |  |  |  |
| No. of centers |  |  |  |
| Single center | 4 (57.1) | 3 (42.9) |  |
| Multicenter | 87 (61.7) | 54 (38.3) |  |
| Region of the coordinating center |  |  |  |
| North America | 42 (59.2) | 29 (40.8) |  |
| Europe | 35 (61.4) | 22 (38.6) |  |
| Asia + MENA | 14 (70.0) | 6 (30.0) |  |
| Funding* |  |  |  |
| Industry | 64 (59.3) | 44 (40.7) |  |
| Public | 13 (68.4) | 6 (31.6) |  |
| Mixed | 11 (64.7) | 6 (35.3) |  |
| Year of start recruitment |  |  |  |
| 1984 - 2003 | 15 (51.7) | 14 (48.3) |  |
| 2004 – 2007 | 19 (54.3) | 16 (45.7) |  |
| 2008 – 2013 | 25 (69.4) | 11 (30.6) |  |
| 2014 – 2020 | 24 (70.6) | 10 (29.4) |  |

*missing data, values do not add up to 148; ˣ17 trials reported median age, which was used as a substitute for mean age in this table. Non-normal data is represented as median [IQR]. Underrepresentation of female patients is defined as PPR < 0.8, adequate representation of female patients is defined as PPR between 0.8 and 1.2.

Abbreviations: ACE, angiotensin-converting enzyme; ARB, angiotensin receptor blockers; CHD, coronary heart disease; JACC, Journal of the American College of Cardiology; JAMA, Journal of the American Medical Association; MENA, Middle East North Africa; NEJM, New England Journal of Medicine; RI, renin inhibitors; SGLT2, sodium-glucose transport protein 2

**Supplementary Table 5: Results from systematic reviews investigating female representation in cardiovascular clinical trials.**

|  | **Disease category** | **Number of studies** | **Outcome measure** | **Key inclusion criteria** | **Key findings** |
| --- | --- | --- | --- | --- | --- |
| Heiat et al, 2002 (22) | HF | 59 | % women | 1985-1999 | - 21% of participants were women - No temporal trend observed - Increased enrollment of women was associated with higher mean age of participants |
| Melloni et al, 2010 (14) | CVD | 156 | % women & sex-stratified results | Drug trials mentioned in 2007 AHA Women’s Prevention Guidelines  1970-2006 | - 30.6% of participants were women - 31% of trials reported sex-stratified results   Proportion of women:   - Increased from 18% in 1970 to 34% in 2006 - Higher in primary versus secondary prevention trials (42.6% versus 26.6%) and in inter thenational versus United States-only trials (32.7% versus 26.7%) - Highest in hypertension (44%), diabetes (40%) and stroke (38%) - Lowest in heart failure (29%), coronary artery disease (25%) and hyperlipidemia (28%) |
| Tsang et al, 2012 (11) | CVD | 325 | Age- and gender-specific disease prevalence | 3 leading journals,  1997-2009 | - 30% of participants were women - No temporal trend observed - Increased female enrollment associated strongly with increasing age at recruitment - Gaps in female enrollment decreased after adjustment for age- and gender-specific differences in prevalence: 5% in coronary artery disease, 9% in arrhythmias and 13% in heart failure |
| Poon et al, 2013 (23) | All diseases | 61 | % women | New molecular entity drugs and biologics approved by FDA, 2007-2009 | - 42% of participants in cardiovascular clinical trials were women - Out of 15 disease categories, cardiovascular trials had relatively poor female participation - A worse score on female participation was observed for trials investigating medical imaging (33%), HIV (12%) and gout (5%) |
| Scott et al, 2018 (12) | CVD | 36 | PPR & sex-stratified safety results | Trials supporting drug approvals submitted to FDA,  2005-2015 | - 46% of participants were women - No temporal trend observed - Trials investigating heart failure (PPR 0.55), coronary artery disease (PPR 0.6) and acute coronary syndrome (PPR 0.6) underrepresented women - Trial investigating pulmonary hypertension (PPR 1.4) overrepresented women - 86% of trials reported sex-stratified safety results - Minimal gender differences in drug efficacy and safety profiles were observed |
| Tahhan et al, 2018 (24) | HF | 118 | % women | 2001-2016 | - 27% of participants were women - No temporal trend observed   Proportion of women:   - Higher in HFpEF (56%) than HFrEF and acute HF (24% and 32%) - Higher in North America (32%) than Western Europe (26%) - Higher in testing nondrug/noninvasive therapies (32%) than drug and invasive therapies (27% and 25%) - Is higher with a higher mean age of participants - Higher in trials with more patients included per site per month (enrollment rate) |
| Whitelaw et al, 2021 (8) | HFrEF | 317 | PPR < 0.8 | IF ≥ 10, 2000-2019 | - 25.5% of participants were women - No temporal trend observed   Factors associated with underrepresentation of female patients:   - Sex-related eligibility criteria (OR 2.05) - Recruitment in in-patient setting (OR 2.56) - Trial coordination in North America (OR 4.44), Europe (OR 6.79) and Asia (OR 9.33) - Drug (OR 1.76) and device/surgery interventions (OR 1.69) - Men in first and last authorship position (OR 1.32) |
| Jin et al, 2020 (3) | CVD | 740 | PPR, female-to-male ratio | 2010–2017 | - 38.2% of participants were women, median female-to-male ratio 0.51 - Trials investigating arrhythmias (PPR 0.78), coronary heart disease (PPR 0.67), stroke (PPR 0.73), acute coronary syndromes (PPR 0.75) and multiple cardiovascular diseases (PPR 0.75) underrepresented women - Trials investigating pulmonary hypertension (PPR 1.33) overrepresented women - All age groups except <55 had a PPR <0.8 - Trials (co)sponsored by government had the lowest PPR - PPR increased between 2013 and 2017 in trials investigating stroke and heart failure - Lowest participation rate (26%) among women aged 61 to 65 years old |
| Steinberg et al, 2021 (10) | All diseases | 20,020 | Female representation relative to corresponding disability-adjusted life years | 2000-2020 | - Out of 17 disease categories, cardiovascular trials had relatively poor female participation - Only pediatric trials scored worse |
| Noubiap et al, 2022 (25) | AF | 142 | Enrollment disparity difference & sex-stratified results | 12 top-tier journals,  2011-2021 | - On average: underenrollment of women by an absolute difference of 12.5 percentage points - Higher enrollment disparity difference in trials with larger sample size (OR 1.065), higher mean age (OR 1.006) and lower enrollment disparity difference in trials conducted in North America (OR 0.945) - 25.4% of trials reported sex-stratified results |

Abbreviations: AF, atrial fibrillation; AHA, American Heart Association; CVD, cardiovascular disease; FDA, Food & Drug Administration; HF, heart failure; HFpEF, heart failure with preserved ejection fraction; HFrEF, heart failure with reduced ejection fraction; IF, impact factor; OR, odds ratio; PPR, participation to prevalence ratio

References in Supplemental Material only

39. Tsao CW, Aday AW, Almarzooq ZI, Alonso A, Beaton AZ, Bittencourt MS, Boehme AK, Buxton AE, Carson AP, Commodore-Mensah Y, Elkind MSV, Evenson KR, Eze-Nliam C, Ferguson JF, Generoso G, et al. Heart Disease and Stroke Statistics—2022 Update: A Report From the American Heart Association. *Circulation*. 2022;145(8).

40. Prins KW, Thenappan T. World Health Organization Group I Pulmonary Hypertension: Epidemiology and Pathophysiology. *Cardiology Clinics*. 2016;34(3):363–374.
